# The Mediating Role of Normal Triglyceride Levels on Lymphocyte Populations in Abdominal Obesity: A Cross‐Sectional Study

**DOI:** 10.1002/hsr2.72098

**Published:** 2026-04-08

**Authors:** Feng Li, Wen‐Jie Wang, Xun Du, Yong Chen, Xiao‐Lin Li, Cheng Song, Fang‐Cen Yuan, Hai‐Qiao Zhang, Xiao‐Chuan Wang, Yun Lu

**Affiliations:** ^1^ Health Management Center Taihu Rehabilitation Hospital of Jiangsu Province Wuxi City P. R. China; ^2^ Department of Clinical Immunology Children's Hospital of Fudan University, National Children's Medical Center Shanghai City P. R. China; ^3^ Shanghai Institute of Infectious Disease and Biosecurity Fudan University Shanghai City P. R. China

**Keywords:** abdominal, lymphocyte subsets, mediation analysis, obesity, triglycerides, waist circumference

## Abstract

**Background and Aims:**

Abdominal obesity is linked to an increased risk of non‐communicable diseases (NCD) and may affect immune function, particularly lymphocyte subpopulations. Waist circumference (WC) is a common indicator of abdominal obesity, but its relationship with lymphocyte subsets is unclear. The role of triglycerides (TG) in this context remains uncertain. This study aims to investigate the relationship between WC and lymphocyte subpopulations, while also evaluating TG's potential mediating effect.

**Methods:**

A retrospective analysis was conducted on physical examination records from March 2021 to September 2023, excluding incomplete data or extreme values. A total of 1836 records with normal TG was analyzed using t‐tests, linear regression, and mediation analysis via SPSS 29.0 software.

**Results:**

After adjusting for confounders, WC was significantly positively correlated with CD3+ T cell count, CD4+ T cell count, and overall lymphocyte count, with TG demonstrating a full mediating effect (Beta [95% CI]: 2.11 [1.39–2.91], 1.39 [0.93–1.89], and 2.83 [1.86–3.87], respectively). WC were also correlated with B cell count, with TG showing a partial mediating effect (Beta [95% CI]: 0.65 [0.44–0.87], with a mediation effect ratio of 30.37%, and 0.02 [0.01–0.02], with a mediation effect ratio of 33.33%).

**Conclusion:**

In health checkups, individuals with normal TG levels, TG plays a significant mediating role in the changes of lymphocyte subpopulations related to abdominal obesity, particularly exerting a positive influence on CD4+ T cells and B cells, with no significant effect on NK cell numbers. These findings provide new insights into the relationship between abdominal obesity and immune dysregulation.

## 1 Introduction

Abdominal obesity, characterized by the excessive accumulation of visceral adipose tissue (VAT), affects nearly 40% of adults worldwide [1, 2]. It poses significant health risks, contributing to an increased prevalence of non‐communicable diseases (NCDs) such as cardiovascular diseases, type 2 diabetes, and metabolic disorders [3, 4, 5]. Beyond its metabolic consequences, abdominal obesity is increasingly recognized for its adverse impact on the immune system, potentially impairing the body's defense mechanisms and promoting chronic low‐grade inflammation [6, 7].

Lymphocyte subpopulations, as key components of the adaptive immune system, play a crucial role in maintaining immune homeostasis. Dysregulation of these populations, including T cells, B cells, and natural killer (NK) cells, has been linked to obesity‐related immune dysfunction and may contribute to the development of obesity‐associated comorbidities [8]. While previous studies have established that abdominal obesity can influence lymphocyte dynamics through various mechanisms [9, 10, 11], the exact pathways and underlying processes remain incompletely understood. While body mass index (BMI) has traditionally been used to assess obesity, it fails to account for differences in fat distribution, which is a key determinant of metabolic risk [12, 13]. In contrast, Waist Circumference (WC), which directly reflects abdominal fat accumulation, is more strongly correlated with visceral fat area (VFA) and has been shown to be a better predictor of cardiovascular risk and metabolic abnormalities [14, 15, 16]. This makes WC a critical parameter for understanding the immunological impacts of obesity.

Triglycerides (TG), a key metabolic marker closely associated with abdominal obesity, may play an important role in the mediating the relationship between abdominal obesity and immune dysfunction. TG serves as a major energy source for the body but is also implicated in metabolic disturbances that can affect immune cell function. Recent studies suggest that TG may influence immune cell activity through various mechanisms, including metabolic reprogramming, lipotoxicity, and alterations in signaling pathways that modulate immune responses [17, 18]. However, the mechanistic evidence for these effects mostly comes from animal models or populations with pathological or metabolic abnormalities (such as those with metabolic syndrome (MetS), dyslipidemia, chronic inflammation, or immune‐related diseases). It remains unclear whether similar immune‐metabolic alterations occur in individuals within the clinically normal TG range (TG < 1.7 mmol/L).

In light of this, the present study aims to explore the relationship between abdominal obesity, as measured by WC, and lymphocyte subpopulations, while evaluating the mediating role of TG within the clinically normal range. By clarifying how TG influences immune cell dynamics in the context of abdominal obesity, this study seeks to provide new insights into early metabolic‐immune interactions and to lay the foundation for potential interventions targeting immune dysfunction in individuals with abdominal obesity.

## 2 Materials and Methods

### 2.1 Study Design

This study utilizes a retrospective, cross‐sectional design to examine the relationship between WC, TG, and lymphocyte subpopulations. Clinical and laboratory data were collected from participants undergoing routine health check‐ups at the Taihu Rehabilitation Hospital of Jiangsu Province. The study was approved by the hospital's Ethics Committee (approval number: YXLL23015). Given the retrospective nature of the study, the Ethics Committee granted a waiver for informed consent, contingent upon the implementation of data anonymization and appropriate privacy protection measures. All analyses were performed in accordance with standardized protocols and ethical guidelines.

### 2.2 Study Population

A total of 3817 participants from the general population, who underwent routine health check‐ups between March 2021 and September 2023, were initially screened. The following exclusion criteria were applied to ensure data quality and minimize confounding:

1. Missing key variables, data or data overlap (580 cases, excluded by Complete Case Analysis).
2. Evidence of acute infections (74 cases);
3. Individuals with white blood cell count < 3.5 × 10^9^/L or lymphocyte count < 1.0 × 10^9^/L (37 cases) or CD4+/CD8+ T‐cell ratio < 0.1 or > 10 (2 cases) [19];
4. Pregnancy (7 cases);
5. Severe liver or kidney dysfunction (157 cases);
6. Malignant tumors, chronic wasting diseases, or other metabolic conditions unrelated to MetS (87 cases);
7. TG ≥ 1.7 mmol/L (1037 cases). Research has shown that elevated TG levels (TG ≥ 1.7 mmol/L) may interfere with certain test results, potentially affecting their accuracy [20].

**Figure 1:**
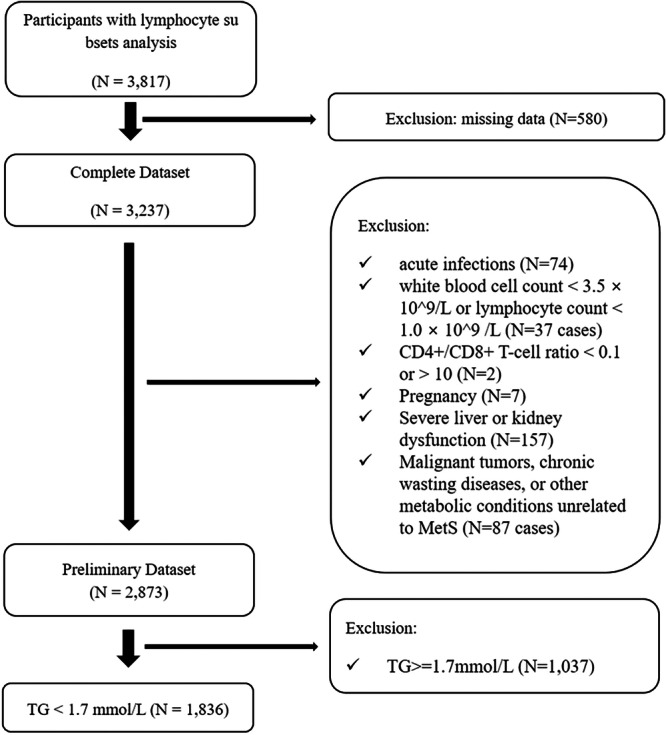
This flow diagram depicts the participant selection process. A total of 3817 individuals were initially screened, with exclusions based on missing data, acute infections, abnormal blood counts, severe health conditions, and TG ≥ 1.7 mmol/L. After applying these criteria, 1836 participants were included in the final analysis.

Ultimately, 1836 cases were included in the analysis. The inclusion and exclusion process was detailed in Figure 1 (STROBE Flow Diagram).

### 2.3 Data Collection and Measurements

The survey was conducted by qualified medical professionals and included: gender, age, and clinical medical history. Health examination items consist of: WC, systolic blood pressure (SBP), diastolic blood pressure (DBP), fasting plasma glucose (FPG), TG, and high‐density lipoprotein cholesterol (HDL‐C).

WC is measured at the midpoint level between the lower edge of the ribs and the anterior superior iliac spine.

Biochemical indicators: After an 8 to 12‐h fast, venous blood (5 mL) was collected from the antecubital vein. The blood samples were allowed to clot and then centrifuged at 3000 rpm for 10 min to separate serum. Serum was separated for the quantification of FPG, HDL‐C, and TG. All assays were performed on a Hitachi LABOSPECT 008 α automated biochemical analyzer using specific reagent kits from Fujifilm Wako Pure Chemical Corporation (Osaka, Japan). The detailed methodologies were as follows:

FPG was measured by the hexokinase/glucose‐6‐phosphate dehydrogenase (G‐6‐PDH) method (l‐Type Glu2, Cat. No. 995‐28304). The intra‐ and inter‐assay coefficients of variation (CV%) for this assay were less than 0.76% and 0.80%, respectively.

HDL‐C was determined by a direct homogeneous method employing an antibody blockade technique (l‐Type HDL‐C, Cat. No. 996‐31904). The intra‐ and inter‐assay CV% for this assay were less than 0.82% and 1.01%, respectively.

TG was quantified by an enzymatic colorimetric method (GPO‐HMMPS method) incorporating a free glycerol blanking step (l‐Type TG M, Cat. No. 995‐27704). The intra‐ and inter‐assay CV% for this assay were less than 0.74% and 1.45%, respectively.

Quality control was performed for each assay using appropriate commercial control sera according to the manufacturer's instructions to ensure analytical performance.

The enumeration of lymphocyte subsets was performed using flow cytometry on a BD FACSCanto™ II system (BD Biosciences, USA) with the BD Multitest™ 6‐Color TBNK reagent.

Peripheral venous blood was collected into K₂‐EDTA anticoagulated tubes and processed within 24 h of collection. According to the manufacturer's instructions, 20 μL of the reagent—containing fluorescently labeled monoclonal antibodies CD3 (FITC, clone SK7), CD16 (PE, clone B73.1), CD56 (PE, clone NCAM16.2), CD45 (PerCP‐Cy5.5, clone 2D1), CD4 (PE‐Cy7, clone SK3), CD19 (APC, clone SJ25C1), and CD8 (APC‐Cy7, clone SK1)—was added to 50 μL of whole blood. After gentle mixing, the samples were incubated in the dark at room temperature for 15 min, followed by red blood cell lysis with 450 μL 1× BD FACS™ lysing solution. The samples were incubated at room temperature in the dark for an additional 15 min. The stained samples were then analyzed on the BD FACSCanto™ II flow cytometer using the BD FACSCanto clinical software, and lymphocyte subsets were gated based on CD45/SSC characteristics and fluorescence compensation settings provided by the manufacturer.

Daily instrument performance was verified using BD Cytometer Setup & Tracking (CS&T) beads, ensuring that the CV% of fluorescence intensity remained within the acceptable range (typically ≤ 2% across all channels). All procedures were conducted in a laboratory accredited under ISO 15189 standards to ensure traceability and analytical reliability.

According to the diagnostic criteria for MetS outlined in the” Guideline for the prevention and treatment of type 2 diabetes mellitus in China (2020 edition)” [21], the following control variables were introduced:

1. Abnormal blood sugar: FPG ≥ 6.1 mmol/L and/or diagnosed with diabetes and under treatment;
2. Abnormal blood pressure: SBP ≥ 130 mmHg and/or DBP ≥ 85 mmHg and/or diagnosed with hypertension and under treatment;
3. Abnormal HDL‐C: fasting HDL‐C < 1.04 mmol/L.
  Additionally, smoking history was included as a control variable [22].

### 2.4 Statistical Analysis

This study was conducted using SPSS software (version 29.0), in conjunction with the Process plugin (version 4.0). Continuous variables were presented as mean ± standard deviation (SD). Independent samples *t*‐test used to analyze.

Using lymphocyte subpopulation counts and percentages as dependent variables, WC as the independent variable, and TG as the mediating variable, with age, gender, smoking history, abnormal blood sugar, abnormal blood pressure, and HDL‐C abnormalities as control variables, model 4 was selected for the analysis of 12 models. The Bootstrap method was utilized to calculate the confidence intervals for the indirect effect of TG. The mediating effect of TG between WC and lymphocyte subpopulations was examined.

Construct 12 linear regression models, with lymphocyte counts and sub‐populations as the dependent variables and TG as the independent variable, adjusting for the same factors as previously mentioned, to analyze the association between lymphocyte subpopulations and TG. Statistical significance was determined with *p < 0.05, **p < 0.01 and ***p < 0.001. The significance level was predetermined (a priori) to ensure the rigor and reproducibility of the analysis.

## 3 Results

A total of 1836 participants aged from 20 to 80 years old, 1013 males and 823 females were included in this study. Among them, 590 (32.1%) were with abdominal obesity, 483 cases (26.3%) with abnormal blood pressure, 208 cases (11.3%) with abnormal blood sugar, 118 cases (6.4%) with abnormal HDL‐C, and 372 cases (20.3%) were smokers. The results indicate that individuals with abdominal obesity have significantly higher levels of SBP, DBP, FPG, and TG compared to those without abdominal obesity. Additionally, the level of HDL‐C is significantly lower in individuals with abdominal obesity. Furthermore, the prevalence of abdominal obesity is higher in males than in females, and among smokers, the rate of abdominal obesity is higher compared to non‐smokers (Table 1).

**Table 1:** Comparison of immunological and metabolic parameters between abdominally obese and non‐abdominally obese individuals.

Variable	Non‐abdominally obesity	Abdominal obesity	*p*‐value
Age (years, SD)	45.22 (12.515)	52.84 (11.955)	< 0.001***
Gender (*n*)			< 0.001***
Male	550	463	
Female	696	127	
SBP (mmHg, SD)	115.05 (15.94)	126.89 (16.19)	< 0.001***
DBP (mmHg, SD)	74.35 (10.51)	80.61 (11.02)	< 0.001***
FPG (mmol/L, SD)	5.14 (0.78)	5.54 (0.95)	< 0.001***
TG (mmol/L, SD)	0.96 (0.33)	1.20 (0.30)	< 0.001***
HDL (mmol/L, SD)	1.56 (0.33)	1.32 (0.27)	< 0.001***
Smoking (*n*)			< 0.001***
Smoker	193	179	
Non‐smoker	1053	411	
Blood Pressure (*n*)			< 0.001***
Normal	1043	310	
Abnormal	203	280	
Blood sugar (*n*)			< 0.001***
Normal	1159	469	
Abnormal	87	121	
HDL‐C (*n*)			< 0.001***
Normal	1200	518	
Abnormal	46	72	
Lymphocyte Count (cells/μL, SD)	2133.63 (564.52)	2223.63 (632.69)	0.002**
CD3+ T Cell Percentage (%, SD)	68.40 (8.15)	66.61 (8.82)	< 0.001***
CD3+ T Cell Count (cells/μL, SD)	1461.94 (435.81)	1479.87 (471.83)	0.423
CD4+ T Cell Percentage (%, SD)	38.60 (7.29)	38.69 (7.71)	0.802
CD4+ T Cell Count (cells/μL, SD)	825.86 (284.87)	857.69 (291.60)	0.027*
CD8+ T Cell Percentage (%, SD)	25.13 (6.71)	23.75 (7.29)	0.001***
CD8+ T Cell Count (cells/μL, SD)	536.50 (203.47)	529.12 (242.81)	0.522
CD4+/CD8+ Ratio	1.69 (0.69)	1.83 (0.81)	< 0.001***
B Cell Percentage (%, SD)	11.76 (4.12)	12.10 (4.61)	0.119
B Cell Count (cells/μL, SD)	252.86 (119.56)	271.63 (138.49)	0.005**
NK Cell Percentage (%, SD)	19.11 (8.26)	20.50 (9.23)	0.002**
NK Cell Count (cells/μL, SD)	403.22 (204.41)	454.60 (248.84)	< 0.001***

Abbreviations: DBP, diastolic blood pressure; FPG, fasting plasma glucose; HDL‐C, high‐density lipoprotein cholesterol; SBP, systolic blood pressure; TG, triglycerides.

* *p* < 0.05

** *p* < 0.01

*** *p* < 0.001.

### 3.1 Comparison of Immunological and Metabolic Parameters between Abdominally Obese and Non‐Abdominally Obese Individuals

Lymphocyte count was higher in the abdominal obesity group (2223.63 [632.69] cells/μL) compared to the non‐abdominal obesity group (2133.63 [564.52] cells/μL), with a *P*‐value of 0.002. Furthermore, the abdominal obesity group exhibited significantly higher CD4+/CD8+ ratio (1.83 [0.81] vs. 1.69 [0.69], *p* < 0.001), CD4+ T cell count (857.69 [291.60] cells/μL vs. 825.86 [284.87] cells/μL, *p* = 0.027), B cell count (271.63 [138.49] cells/μL vs. 252.86 [119.56] cells/μL, *p* = 0.005), NK cell percentage (20.50 [9.23]% vs. 19.11 [8.26]%, *p* = 0.002), and NK cell count (454.60 [248.84] cells/μL vs. 403.22 [204.41] cells/μL, *p* < 0.001).

Conversely, CD3+ T cell percentage (66.61 [8.82]% vs. 68.40 [8.15]%, *p* < 0.001) and CD8+ T cell percentage (23.75 [7.29]% vs. 25.13 [6.71]%, *p* = 0.001) were significantly lower in the abdominal obesity group.

No significant differences were found between the two groups in CD3+ T cell count (*p* = 0.423), CD4+ T cell percentage (*p* = 0.802), CD8+ T cell count (*p* = 0.522), and B cell percentage (*p* = 0.119) (Table 1).

### 3.2 Mediating Role of TG in the Correlation between WC and Lymphocyte Counts and Subsets

After adjusting for age, gender, smoking history, abnormal blood pressure, abnormal blood sugar, and HDL‐C, the CD3+ T cell count, CD4+ T cell count, and lymphocyte count remained correlated with WC. TG was found to have a complete mediating effect, with mediation effect values [Beta (95%CI)] of 2.11 (1.39–2.91), 1.39 (0.93–1.89), and 2.83 (1.86–3.87), respectively. The B cell count and B cell percentage were also correlated with WC, with TG having a partial mediating effect. The mediation effect values [Beta (95%CI)] were 0.64 (0.44–0.86) and 0.02 (0.01–0.02), respectively, with mediation effect percentages of 30.05% and 33.33%. (For more details, see Table 2).

**Table 2:** Analysis of the mediating effect of TG on the relationship between lymphocyte subpopulations and WC [Beta (95%CI)].

Lymphocyte component	Total effect	Direct effect	Indirect effect	Percentage of total effect mediated by indirect effect
Lymphocyte Count (cells/μL)^1^	5.70 (2.29 to 9.11)*	2.87 (−0.63 to 6.37)	2.83 (1.86 to 3.87)*	49.65% (2.83/5.70)
NK Cell Percentage	−0.05 (−0.10 to 0.00)			
NK Cell Count (cells/μL)	0.24 (−1.04 to 1.53)			
B Cell Percentage	0.06 (0.04 to 0.09)*	0.05 (0.02 to 0.07)*	0.02 (0.01 to 0.02)*	33.33% (0.02/0.06)
B Cell Count (cells/μL)	2.13 (1.40 to 2.86)*	1.49 (0.74 to 2.23)*	0.64 (0.44 to 0.86)*	30.05% (0.64/2.13)
CD3+ T Cell Percentage	−0.01 (−0.06 to 0.04)			
CD3+ T Cell Count (cells/μL)^1^	3.33 (0.76 to 5.90)*	1.22(−1.43 to 3.86)	2.11 (1.39 to 2.91)*	63.36% (2.11/3.33)
CD4+ T Cell Percentage	0.02 (−0.03 to 0.06)			
CD4+ T Cell Count (cells/μL)^1^	2.29 (0.61 to 3.97)*	0.90 (−0.83 to 2.62)	1.39 (0.93 to 1.89)*	60.70% (1.39/2.29)
CD4+/CD8+ Ratio	0.00 (−0.00 to 0.01)			
CD8+ T Cell Percentage	−0.03 (−0.07 to 0.01)			
CD8+ T Cell Count (cells/μL)	0.69 (−0.55 to 1.94)			

*Note:* Control variables include age, gender, smoking history, abnormal blood pressure, abnormal blood sugar, and HDL‐C abnormalities.

Abbreviations: TG, triglycerides; HDL‐C, high‐density lipoprotein cholesterol.

1 The mediation effect is considered complete.

* *p* < 0.05.

### 3.3 TG Influence on Lymphocyte Subpopulations: A Multivariate Linear Regression Analysis

Twelve linear regression models were constructed. These models used lymphocyte counts and sub‐populations as the dependent variables, with TG as the independent variable. The models were adjusted for age, gender, smoking history, abnormal blood pressure, abnormal blood sugar, and HDL‐C abnormalities. The results suggest that TG has a significant positive influence on lymphocyte counts, CD3+ T cell counts, CD4+ T cell percentages, CD4+ T cell counts, CD8+ T cell counts, B cell percentages, and B cell counts, with regression coefficients of 274.343, 198.803, 1.173, 131.61, 53.939, 1.639 and 67.669, respectively, all with *p* < 0.05. Additionally, TG has a significant negative effect on NK cell percentages, with a regression coefficient of −2.282, *p* = 0.014.

Standardized Beta coefficients were used to assess the influence of various immune cell subtypes on the outcome variable. The analysis indicated that TG had a significant positive effect on B cell count, lymphocyte count, CD4+ T cell count, CD3+ T cell count, and CD8+ T Cell Count, with B cell count showing the strongest positive associations. Additionally, TG exhibited moderate positive effects on B cell percentage and CD8+ T cell count. In contrast, TG had a notable negative impact on NK cell percentage (Table 3).

**Table 3:** Multivariate linear regression analysis with lymphocyte subpopulations as dependent variables and TG as the independent variable.

Model	Dependent variable	Standardized beta	Beta	95%CI	*p*‐value
1	Lymphocyte Count (cells/μL)	0.159	274.343	193.079 to 355.606	0.000***
2	NK Cell Percentage	−0.090	−2.282	−3.459 to −1.105	0.014*
3	NK Cell Count (cells/μL)	0.009	5.708	−25.216 to 36.631	0.717
4	B Cell Percentage	0.131	1.639	1.035 to 2.243	0.000***
5	B Cell Count (cells/μL)	0.183	67.669	50.255 to 85.084	0.000***
6	CD3+ T Cell Percentage	0.025	0.615	−0.558 to 1.789	0.304
7	CD3+ T Cell Count (cells/μL)	0.152	198.803	137.512 to 260.094	0.000***
8	CD4+ T Cell Percentage	0.054	1.173	0.139 to 2.208	0.026*
9	CD4+ T Cell Count (cells/μL)	0.156	131.61	91.585 to 171.634	0.000***
10	CD4+/CD8+ Ratio	0.031	0.066	−0.037 to 0.169	0.21
11	CD8+ T Cell Percentage	−0.029	−0.584	−1.566 to 0.398	0.244
12	CD8+ T Cell Count (cells/μL)	0.085	53.939	24.046 to 83.833	0.000***

Adjusted for age, gender, smoking history, abnormal blood pressure, abnormal blood sugar, and HDL‐C abnormalities.

Abbreviations: TG, triglycerides; HDL‐C, high‐density lipoprotein cholesterol.

* *p* < 0.05

*** *p* < 0.001.

## 4 Discussion

### 4.1 Metabolic‐Immune Characteristics of Abdominal Obesity

WC is a scientifically validated and effective non‐invasive measure for assessing abdominal obesity, and it is closely associated with VAT. Although CT is considered the gold standard for assessing abdominal fat distribution, it is not suitable for continuous monitoring or large‐scale studies due to its high cost, the need for consistent interpretation by trained personnel, and the radiation exposure involved. In contrast, WC is an economical, convenient, and reliable measure, making it well‐suited for studying the relationship between abdominal fat and health [23, 24]. The results of our study indicate that individuals with abdominal obesity (men ≥ 90 cm, women ≥ 85 cm) exhibit significant metabolic differences and significant changes in absolute counts of lymphocyte subpopulations compared to those without abdominal obesity.

#### 4.1.1 Metabolic Characteristics

Individuals with abdominal obesity had significantly higher TG levels, FPG, and lower HDL‐C levels compared to those without abdominal obesity. These findings are consistent with the study by Kashihara et al., which linked VAT accumulation to dyslipidemia, impaired glucose regulation, and increased cardiovascular risk [25].

#### 4.1.2 Immune Characteristics

The abdominal obesity group showed significantly higher lymphocyte count, CD4+ T cell count, CD4+/CD8+ ratio, B cell count, NK cell percentage, and NK cell count compared to the non‐abdominal obesity group. In contrast, the abdominal obesity group had significantly lower CD3+ T cell percentage and CD8+ T cell percentage. Changes in the percentage distribution of lymphocyte subpopulations may correlate with alterations in their absolute counts.

Rodríguez et al. [7] observed increased total lymphocytes and memory T cells with rising VAT, which aligns with our finding of an expanded T and B cell pool in individuals with abdominal obesity. Similarly, Prechtl et al. [26] found VAT‐related remodeling of B cell subsets, reinforcing the idea that visceral fat accumulation impacts adaptive immunity. Our results similarly show increased CD4+ T cells and B cells in individuals with abdominal obesity.

However, a divergence exists regarding NK cells. Rodríguez et al. [7] reported reduced NK cell counts in individuals with metabolic syndrome, whereas we found a significant increase in NK cells in the abdominal obesity group. This difference may stem from the unique nature of our cohort, which specifically targeted a normolipidemic subgroup (TG < 1.7 mmol/L). By isolating abdominal obesity from dyslipidemia, our findings suggest that VAT triggers compensatory immune activation—evidenced by increased NK cells—during the early lipid‐normal phase of obesity, prior to the immune exhaustion observed in more advanced metabolic disorders.

Despite this divergence, our study's alignment with general immunometabolic patterns observed in DXA‐based research supports the validity of WC as a reliable surrogate marker for VAT. While CT remains the gold standard, WC provides a robust, practical alternative for large‐scale studies. Moreover, our study advances the field by examining these associations in a lipid‐normal context, emphasizing VAT's independent role in adaptive immune regulation, free from the confounding effects of hypertriglyceridemia.

### 4.2 TG as a Biochemical Link between Abdominal Obesity and Immune Dynamics

This study is the first to identify TG as a critical mediator between abdominal obesity and immune alterations. Mediation analysis revealed that after adjusting for age, sex, smoking history, abnormal blood pressure, impaired blood glucose, and HDL‐C levels, TG played a full mediating role in the association between WC and CD3+ T cell count, CD4+ T cell (CD4+) count, and lymphocyte count. Additionally, TG acted as a partial mediator in the relationship between WC and B cell count, as well as B cell percentage.

These findings suggest that in individuals with normal TG levels (< 1.7 mmol/L), changes in CD3+ T cell count, CD4+ T cell count, and lymphocyte count in relation to WC may be fully mediated by TG. Furthermore, the variations observed in B cell count and B cell percentage may be partially mediated by TG. This implies that the changes in T cell counts in individuals with abdominal obesity are more closely related to TG levels. Even within the normal TG range, TG plays a key role in the immune cell distribution changes associated with abdominal obesity. This highlights TG as a potential key molecular mediator at the intersection of metabolism and immune regulation.

Emerging evidence suggests that abdominal obesity influences lymphocyte dynamics through various mechanisms, such as visceral fat‐induced macrophage infiltration, which promotes a proinflammatory environment and disrupts lymphocyte recruitment and differentiation [9]. Additionally, obesity‐related metabolic changes, including T‐cell metabolic reprogramming, shift T‐cells toward glycolysis rather than fatty acid oxidation, leading to T‐cell exhaustion [10, 11]. TG and their metabolites, including free fatty acids, can activate inflammatory pathways (e.g., TLR4–NF‐κB) and induce metabolic stress [10], further impairing lymphocyte function. However, most of these studies are based on pathological conditions or populations with metabolic abnormalities [17, 18], focusing on the negative impact of elevated TG levels on immune function. In contrast, our study emphasizes TG as a key mediator in the relationship between abdominal obesity and immune alterations, even at normal TG levels.

### 4.3 TG's Immunomodulatory Role in Individuals with Normal Lipid Levels

This study reveals, for the first time, the immunomodulatory role of TG in individuals with normal lipid levels (< 1.7 mmol/L). Unlike previous research focused on hypertriglyceridemic states, we demonstrate that even normal‐range TG significantly impacts immune system dynamics. After adjusting for confounding factors, TG levels showed significantly positive correlations with multiple T cell subpopulations and B cell counts, altering their distribution ratios. These findings further contribute to our understanding of TG's immune‐modulating effects in non‐pathological conditions.

Previous studies exploring the relationship between TG and immune cells have reported inconsistent findings. Xu et al. [27] investigated lymphocyte subsets in peripheral blood from patients with lipid abnormalities but found no significant association between TG levels and lymphocyte populations. This result may have been limited by their small sample size of 51 hyperlipidemic patients. On the other hand, Liu et al. [28] reported that in a specific population of recurrent pregnancy loss (RPL) patients without polycystic ovary syndrome (PCOS) or obesity, elevated TG levels were associated with an increased CD3+ CD4+/CD3+ CD8+ ratio.

Recent mechanistic studies provide deeper insights into how TG mediates these immune effects. At the molecular level, TG and their metabolites directly regulate immune cell function through multiple interconnected pathways. For T cells, lipid metabolism reprogramming is fundamental to T cell differentiation and function [29, 30]. TG promotes Th1 differentiation [31] and supports B cell activation and antibody production [32]. Additionally, fatty acid oxidation supports tissue‐resident memory T cell survival [33]. Moreover, TG‐derived fatty acids provide the necessary ATP and biosynthetic precursors for T and B cell expansion, explaining the positive correlation we observed between TG levels and lymphocyte counts [33, 34]. For B cells, TG serves as both an energy source and a signaling molecule, promoting expression of activation markers and facilitating differentiation toward antibody‐producing plasma cells [35, 36].

These mechanisms might explain why normal‐range TG exerts significant immunomodulatory effects: immune cells are exquisitely sensitive to metabolic fluctuations that appear minor systemically but are substantial at the cellular level [37].

### 4.4 Clinical Applications of Immune Resilience

Immune resilience refers to the ability of the immune system to maintain or restore functional stability in the face of inflammation or metabolic stress [38]. This study suggests that TG may play a significant role in regulating adaptive immunity, thereby influencing immune resilience under metabolic stress conditions. Specifically, changes in TG levels in our study were associated with adaptive immune functions. TG showed a significant positive correlations with lymphocyte count, CD3+ T cell count, CD4+ T cell count, CD8+ T cell count, and B cell count, suggesting that TG may help maintain immune system stability by modulating adaptive immune function.

According to the study [38], immune health grades (IHG) are defined based on CD4+ T cell count and the CD4:CD8 ratio. Specifically, IHG‐I (the optimal immune health state) is defined as a CD4+ T cell count ≥ 800 cells/mm³ and a CD4:CD8 ratio ≥ 1.0, representing an immune system in equilibrium with high resilience.

To further evaluate the relationship between TG and immune health, a logistic regression model was established with immune health status as the dependent variable and TG (standardized with age, gender, smoking history, abnormal blood pressure, abnormal blood sugar, and HDL‐C abnormalities) as the predictor. The model demonstrated a moderate discriminative ability (AUC = 0.61), with a positive regression coefficient for TG (β = 0.253), indicating that higher TG levels were associated with a greater likelihood of optimal immune health. The predicted probability of IHG‐I reached 0.5 when TG was approximately 1.20 mmol/L. As TG levels increased, the prevalence of optimal immune health progressively rose—from 43.6% (TG ≤ 1.0 mmol/L) to 52.7% (TG ≈ 1.7 mmol/L). However, the overall model accuracy was 59%, suggesting that TG alone may not serve as a sufficient predictor of immune health.

It can be further speculated that when TG levels are too low, adaptive immune function (such as CD4+ T cell count and B cell activity) may be suppressed, thereby compromising overall immune resilience. Maintaining moderately elevated TG levels, particularly above 1.2 mmol/L, may therefore be critical for preserving immune homeostasis and resilience. Future studies should aim to identify the optimal TG threshold that balances metabolic health and immune stability, and to investigate whether TG could serve as a potential target for modulating immune cell equilibrium.

Most previous studies have primarily emphasized the negative impact of elevated TG levels on immune function, as discussed earlier. However, beyond these adverse effects, it is crucial to recognize that immune cells also rely on lipid metabolism to provide essential energy and signaling molecules for their survival and function. TG and its metabolites play a pivotal role in regulating immune cell activity by promoting T cell differentiation, B cell activation, and supplying the necessary energy and biosynthetic precursors for immune cell expansion [33, 36, 37]. The threshold effect observed likely represents the minimum metabolic requirements needed for immune cells to maintain baseline function and effectively respond to immune challenges.

Future studies should further explore the optimal TG threshold that balances metabolic health and immune stability, and investigate whether TG could serve as a potential target for modulating immune cell equilibrium. However, these results should be interpreted with caution due to the observational nature of the study, and prospective research is needed to validate these findings.

### 4.5 Dynamic Interactions and Causal Relationships

Our findings align with a recent Mendelian randomization study [39], which revealed a bidirectional causal relationship between TG levels and immune cell counts. Specifically, elevated TG levels were causally linked to increases in lymphocyte and neutrophil counts, while elevated lymphocyte levels, in turn, were predictive of further increases in TG levels.

This dynamic interaction underscores the complex interplay between lipid metabolism and immune regulation, further highlighting TG's dual role as both a metabolic marker and an immune regulatory factor. Unfortunately, there have been no Mendelian randomization studies investigating the relationship between lymphocyte subpopulations and TG levels.

Nevertheless, the bidirectional causal relationship between TG levels and immune cell counts might further validate our previous notion: lipid metabolism plays a dual role in immune cell function, exerting both negative effects as well as supporting the normal physiological functions of immune cells. This duality requires a nuanced, bidirectional approach to understanding their relationship, emphasizing the importance of considering both the beneficial and detrimental impacts of lipid metabolism on immune regulation. Our interpretation of the relationship between TG levels and immune cells must therefore be viewed through this complex, dynamic lens.

### 4.6 Study Limitations and Future Research Directions

This study has several limitations. Firstly, the cross‐sectional design restricts causal inference, highlighting the need for future prospective cohort studies. Secondly, our sample included only individuals with TG levels below 1.7 mmol/L, which limits the generalizability of our findings to populations with higher TG levels. Third, unmeasured factors such as dietary habits, physical activity, and genetic predisposition may have influenced the observed associations. Fourth, we did not account for the use of lipid‐lowering medications, which could also impact the results.

Building on the findings of this study, future research could further analyze lymphocyte differentiation using flow cytometry to determine whether the increase in lymphocyte count is due to the expansion of effector cells, memory cells, or senescent cells. Additionally, research on TG levels could explore the optimal threshold, as well as the effects of pharmacological interventions and lifestyle changes. Prospective studies are needed to assess the long‐term impact of these factors on TG levels and immune function.

## 5 Conclusion

This study reveals the immune characteristics of abdominal obesity and highlights the key role of TG in the relationship between abdominal obesity and lymphocyte counts and subsets. For the first time, we demonstrate the regulatory effect of TG on the immune system in individuals with normal lipid levels. TG not only reflects changes in lipid levels but also regulates immune regulation by altering the absolute counts and functional responses of immune cells. Our findings suggest the importance of balancing metabolic health and immune resilience. Future research should further explore the optimal TG threshold and its mechanisms to inform personalized interventions.

## Author Contributions

**Feng Li:** conceptualization, methodology, formal analysis, funding acquisition, investigation, writing – original draft, validation. **Wen‐Jie Wang:** conceptualization, methodology, formal analysis, writing – original draft, critical revision. **Xun Du:** data curation, investigation, writing – original draft. **Yong Chen:** data curation. **Xiao‐Lin Li:** data curation. **Cheng Song:** investigation. **Fang‐Cen Yuan:** data curation. **Hai‐Qiao Zhang:** Validation. **Xiao‐Chuan Wang:** conceptualization, methodology, formal analysis, critical revision. **Yun Lu:** conceptualization, methodology, formal analysis, investigation, funding acquisition, critical revision.

## Ethics Statement

The study was approved by the Ethics Committee of the Taihu Rehabilitation Hospital of Jiangsu Province (approval number: YXLL23015). Given the retrospective nature of the study, the Ethics Committee granted a waiver for informed consent, contingent upon the implementation of data anonymization and appropriate privacy protection measures. All analyses were performed in accordance with standardized protocols and ethical guidelines.

## Conflicts of Interest

The authors declare no conflicts of interest.

## Transparency Statement

The lead authors, Yun Lu and Xiao‐Chuan Wang affirm that this manuscript is an honest, accurate, and transparent account of the study being reported; that no important aspects of the study have been omitted; and that any discrepancies from the study as planned (and, if relevant, registered) have been explained.

## Data Availability

The datasets used to support the findings of this study are available from the corresponding author upon reasonable request.
